# Iodisation of Salt in Slovenia: Increased Availability of Non-Iodised Salt in the Food Supply

**DOI:** 10.3390/nu8070434

**Published:** 2016-07-16

**Authors:** Katja Žmitek, Igor Pravst

**Affiliations:** 1 Nutrition Institute, Tržaška cesta 40, Ljubljana SI-1000, Slovenia; katja.zmitek@vist.si; 2 VIST—Higher School of Applied Sciences, Gerbičeva cesta 51A, Ljubljana SI-1000, Slovenia

**Keywords:** salt, sodium, iodine, sales, food supply, Slovenia

## Abstract

Salt iodisation is considered a key public health measure for assuring adequate iodine intake in iodine-deficient countries. In Slovenia, the iodisation of all salt was made mandatory in 1953. A considerable regulatory change came in 2003 with the mandatory iodisation of rock and evaporated salt only. In addition, joining the European Union’s free single market in 2004 enabled the import of non-iodised salt. The objective of this study was to investigate the extent of salt iodising in the food supply. We examined both the availability and sale of (non-)iodised salt. Average sales-weighted iodine levels in salt were calculated using the results of a national monitoring of salt quality. Data on the availability and sales of salts were collected in major food retailers in 2014. Iodised salt represented 59.2% of the salt samples, and 95.9% of salt sales, with an average (sales-weighted) level of 24.2 mg KI/kg of salt. The average sales-weighted KI level in non-iodised salts was 3.5 mg KI/kg. We may conclude that the sales-weighted average iodine levels in iodised salt are in line with the regulatory requirements. However, the regulatory changes and the EU single market have considerably affected the availability of non-iodised salt. While sales of non-iodised salt are still low, non-iodised salt represented 33.7% of the salts in our sample. This indicates the existence of a niche market which could pose a risk of inadequate iodine intake in those who deliberately decide to consume non-iodised salt only. Policymakers need to provide efficient salt iodisation intervention to assure sufficient iodine supply in the future. The reported sales-weighting approach enables cost-efficient monitoring of the iodisation of salt in the food supply.

## 1. Introduction

Iodine is an essential component of thyroid hormones and plays a key role in regulating human growth, development, and metabolic functions. Its sufficient dietary intake is crucial for preventing iodine deficiency disorders such as goitre, neurocognitive impairment, hyperthyroidism, and hypothyroidism [1,2]. While goitre is the most visible consequence of iodine deficiency, the major impact of iodine-deficiency related hypothyroidism is impaired neurodevelopment, particularly during fetal and early postnatal life [3,4]. Inadequate thyroid hormone impairs myelination, cell migration, differentiation, and maturation in the fetal brain, and iodine deficiency during pregnancy increases rates of spontaneous abortion, reduces birth weight, and increases infant mortality [3]. A growing body of evidence shows that post-natal cognitive development is adversely affected not only in moderate-to-severe, but also in mild-to-moderate prenatal iodine deficiency [5]. Iodine deficiency also affects the intellectual development of infants and children; the damage increases with the extent of the deficiency, with overt endemic cretinism as the severest consequence, combining irreversible mental retardation, neurological damage, and thyroid failure [4].

The iodine content of local foods depends on soil iodine content and, therefore, low iodine concentrations in soil and water result in iodine-deficient plants and animals. Consequently, humans that depend on food sources grown in iodine-deplete or iodine-deficient areas become iodine deficient in the absence of any other source of this micronutrient [1].

In most developed countries where populations were previously at risk for iodine deficiency, the main source of iodine is iodised salt and salt iodisation is considered a key public health measure for preventing thyroid disorders.

In Slovenia, in the first half of the 20th century the estimated goitre prevalence was 58% among schoolchildren [6], and up to 80% in the general population [7]. Mandatory iodisation of all table salt was introduced in 1953 at the level of 10 mg KI per kilogram of salt. This led to a drop in the prevalence of goitre in school children to 22% in 1959 [6], and to 12% in 1991 [7,8]. In 1999 the mandatory degree of iodising all table salt was increased to 25 mg of KI/kg [9], resulting in a change from a mildly deficient to a sufficient iodine supply in Slovenia. A ten-year follow-up showed a notable alteration in the epidemiology of thyroid disorders with a gradual drop in the incidence of diffuse goitre [10]. 

A considerable regulatory change came in 2004 when Slovenia joined the European Union (EU), and a new national regulation on salt was accepted [11]. This regulation provided the mandatory iodisation of rock salt and evaporated salt (water extracted from underground deposits, the water is then evaporated using a heat process in a vacuum evaporator), but not for the most common sea salt. However, it should be noted that this national regulation was not notified to the EU, where salt iodisation is not mandatory and, therefore, Slovenian authorities do not have legal grounds to restrict the sale of non-iodised salt, which is legally sold in any other EU Member State. The reason for this lies in the single EU market, entailing the free movement of goods and services, as defined in the Treaty on the Functioning of the European Union (TFEU) [12]. Any national regulations which limit the single market (including the free movements of foods) are regarded as trade barriers. According to Article 36 of the TFEU, such exceptions are only possible when justified by strict non-economic considerations, while respecting the principle of proportionality. Protection of human health is seen as a justifiable national exemption. EU Member States have to notify national exemption measures to the European Commission and procedures for the exchange of information [13]. In such a notification procedure, an EU Member State needs to provide evidence to justify the national exemption. 

While in the initial years after joining the EU such referrals to the free movements of goods were not made very often, the situation changed over time. In 2013, authorities published a monitoring report showing that several non-iodised salts were available in the Slovenian food supply [14]. Reports on the growing availability of non-iodised salt were also made in some other iodine-deficient countries [15], implying a concern for adequate iodine intakes in the population. However, a recent analysis of the US market shows the proportion of available non-iodised brands of salt does not necessarily correlate with the sales levels; although only 5% of available salts was iodised, they represented 53% of total sales [16].

The objective of our study was to investigate the extent of salt iodising in the food supply in Slovenia. We investigated both the availability and sales of (non)iodised salt. Average sales-weighted iodine levels in salt were calculated using the results of a national monitoring of salt quality.

## 2. Experimental Section 

### 2.1. Collection of Data

Data on the (labelled) composition of salt products were collected in four grocery stores in the capital, Ljubljana (two megamarkets, two supermarkets). To ensure the sample’s high representativeness, we selected grocery stores of retailers with accessible nation-wide store networks and the largest market shares. These retailers accounted for over 50% of the total national market share in terms of sales value, and operated in all parts of the country. The database was composed in agreement with the retailers where the data were collected. In the next stage, the retailers were asked to provide 12-month, country-wide sales data for each salt product sold (January 2014–December 2014). The final sample was composed of 86 salts and salt blends. The sales data were given in universal form, including the European/International Article Number (EAN barcode), description of the product, number of products sold per year, and the quantity of food (kg) per packaging. The matching of foods between the databases was performed using EAN barcodes. Based on the name of the product and/or ingredients, all samples were categorised as: (a) iodised salt; (b) non-iodised salt; or (c) salt blends (e.g., garlic salt, herbal salt). Samples were further sub-categorised into sea salt (including gourmet sea salt) and rock salt (including Himalayan salt), and as shaker packets (salt in small-volume dispensers (shaker, grinder) designed for table use). The sample did not include mixtures of sodium and potassium chloride.

Data on the iodine content in the salt samples (excluding salt blends) were assigned using results of the national monitoring of salt quality in Slovenia conducted by the Administration of the Republic of Slovenia for Food Safety, Veterinary and Plant Protection (Ministry of Agriculture and the Environment, Slovenia) [14]. The monitoring was performed in 2013; iodine content was determined by the National Laboratory of Health, Environment and Food, Slovenia (NLZOH), using a standard procedure [17]. Considering yearly sales values, we were able to match iodine levels for 78% of the salt in our database. The remaining salt samples (for which we did not find a comparable match in the monitoring programme) were assigned with average within-category iodine content. 

### 2.2. Data Analyses 

Availability of (non-)iodised salt samples was calculated as a proportion of (non-)iodised samples in: (a) the whole sample of salts; and (b) the whole sample of salts excluding the salt blends. Proportion of total salt sales was calculated for the selected (sub)categories, considering 12-month sales data on the national level (in kg per year). The calculation was again performed for both the whole sample and with exclusion of the salt blends. Iodine content was calculated for (non-)iodised salt, and sea/rock salt, using a sales-weighting approach [18,19]. The sales data the retailers provided were combined to calculate the number of products sold per year for each food product in the database. Using data on the content of food per packaging, we calculated the amounts (kg) of products sold per year. In the next stage, the total content of iodine in salts sold in a specific category (kg) was calculated using the assigned iodine levels. Finally, average iodine content in the (sub)category was calculated in mg of KI per kg of salt. Analyses were performed using Microsoft Excel 2013 (Microsoft Corporation, Redmond, WA, USA). 

## 3. Results 

In total, 86 salt products were identified; the availability of (non-)iodised salt samples is presented in Table 1. We identified 42 iodised salts (48.8%, among which 37.2% were sea salts and 11.6% rock salts), 29 non-iodised salts (33.7%: 12.8% sea salt and 20.9% rock salt), and 15 salt blends (17.4%). Although the salt-blend samples represented a notable proportion of the sample, their total sales share was very low (0.2%) and were, therefore, excluded from further analyses. In the remaining sample, iodised salt represented 59.2% of the available salt samples, and 95.9% of the salt sales, with an average (sales-weighted) level of 24.2 mg KI per kg of salt (interval: 20.9–45.8 mg KI/kg). Average sales-weighted KI levels in the non-iodised salts (representing 4.1% of the sales volume) were much lower (3.5 mg KI/kg; interval: 1.3–5.2 mg KI/kg). 

## 4. Discussion 

Iodised salt is the most important dietary source of iodine in Slovenia [20]. Mandatory iodisation of salt has been correlated with a notable decrease in the incidence of diffuse goitre in Slovenia [10], making it one of the key national public health interventions. Although it has been shown that the majority of the population has a sufficient iodine intake, reported case studies indicate that persons with alternative dietary habits, whereby they do not consume iodised salt, are still at risk of iodine deficiency [21,22].

According to the national regulation on salt [11], only evaporated and rock salt must be fortified with 25 mg of KI/kg, with a tolerance interval 20–30 mg. While it has not been mandatory to fortify sea salt since 2003, we determined that 96% of sea salt that is sold is actually still iodised. This is particularly important because sea salt represents the vast majority (86.1%) of salt sold in the market. Considering that iodine levels in non-iodised sea salt were several times lower than the target iodisation levels, there is no rationale to exempt sea salt from the mandatory iodisation.

Results of this study also show that the regulatory changes in 2003, and the lack of the implementable mandatory fortification of salt, have led to the considerable availability of non-iodised salt. We determined that one-third of the available salts is non-iodised, but these products still account for low sales levels. While these results suggest an efficient salt-derived iodine supply for the majority of the population (currently mostly on the basis of voluntary fortification of sea salt), the notable proportion of non-iodised salts strongly indicates the existence of a niche market which could pose a risk of inadequate iodine intake in those who deliberately decide to consume non-iodised salt only, and also avoid the consumption of processed foods, which are also an important source of salt and iodine [18]. A relevant question is how to assure a sufficient iodine supply not only for the majority, but for the whole population. As stated by Sankar et al., “there are situations in which, in the absence of proper education, “the freedom to choose” may not offer the right choice, and salt iodization is one of them” [23]. In such a case, an efficient regulation is essential and needs to be combined with advocacy, consumer education, and quality assurance [23]. While the mandatory fortification of salt is clearly the most efficient public-health intervention, the question remains of whether such an intervention is actually enough to protect everyone. In the Internet era, where foods can easily be purchased online, consumers seeking non-iodised salt can simply order it from abroad. It should be noted that experts in Slovenia are strongly promoting the consumption of iodised salt, but contrary opinions are also available online and in alternative media. 

A promising decision to resolve the above-mentioned issues was accepted by the Slovenian government, which proposed a new national regulation on salt. The proposed regulation provides for the mandatory fortification of all table salt (including sea salt), and define a special requirement for the labelling and marketing of non-iodised salt. All non-iodised salts (and store shelves on which such products are displayed) must be equipped with the statement “Salt is not enriched with iodine, which is essential for normal thyroid function”. The proposed regulation also provides marketing prohibitions on non-iodised salt, including a prohibition on offering samples, advertising, distance selling, special offers, and discount coupons.

A major strength of this study is its employment of academic-commercial collaboration which enabled the use of the sales-weighting approach. However, this is also connected with the main limitation, namely that our sample did not cover the complete national market. Given that the study was performed by including major retailers with accessible nation-wide store networks, which accounted for over 50% of the total national market share in terms of sales value, we believe that the result reported herein represents a very good estimate for the whole Slovenia market. A better approach would be to use commercially available sales data, as was done by Maalouf et al. for the US market [16], but such an approach is related with considerable costs which, in our case, made it unfeasible. Another limitation of the study is that the assessment of iodine content was done using the results of monitoring collected in 2013, while our study was performed for salts sold in 2014, and that iodine levels were not available for all samples. However, no regulatory or other notable changes occurred on the market between 2013 and 2014. Further, we were able to assign iodine levels to over three-quarters of the salt, while average within-category iodine content was assigned for the remaining 22% of the salt. Another important limitation is that our study only provides data for retailers with nation-wide store networks. People who choose an alternative lifestyle may see iodine as an unnecessary additive, and may also deliberately avoid large supermarkets, preferring specialised shops with “more natural” food. Therefore, future research should also focus on investigating such a specialised retail environment.

## 5. Conclusions 

Sales-weighted average levels of iodine in iodised salt are in line with the regulatory requirements. The results reported herein show that the regulatory changes in 2003 and the EU single market have resulted in the increased availability of non-iodised salt, which represented 33.7% of the salt samples, but sales levels of those products are still low. While the results suggest an efficient salt-derived iodine supply for the majority of the population, it should be noted that it is primarily based on voluntary fortification of sea salt. However, while sales of non-iodised salt are still very low, the above-mentioned proportion of non-iodised salts strongly indicates the existence of a niche market which could pose a risk of inadequate iodine intake in those who deliberately decide to consume non-iodised salt only. Policy-makers need to ensure a sufficient iodine supply in the population. Salt iodisation has been revealed to be a very efficient public health intervention, which should be accompanied with additional actions to assure the education of consumers on the importance of sufficient dietary intake of iodine. The reported approach enables the cost-efficient monitoring of the iodisation of salt in the food supply.

## Figures and Tables

**Table 1:** Availability of (non-)iodised salt, proportion of total salt sales and iodine content by type of salt (Slovenia, 2014).

Salt Type	All Samples			Samples Excluding Salt Blends				
	*N* ^1^	*N* (%)	Sales ^2^	*N* ^1^	*N* (%)	Sales ^2^	mg KI/kg	
							Interval ^3^	Weighted Average ^4^
**Iodised salt**	**42**	**48.8%**	**95.7%**	**42**	**59.2%**	**95.9%**	**20.9–45.8**	**24.2**
Sea salt	32	37.2%	82.7%	32	45.1%	82.9%	28.8–45.8	24.2
as Shaker packet	10	11.6%	0.6%	10	14.1%	0.6%	-	-
Rock salt	10	11.6%	13.0%	10	14.1%	13.0%	20.9–36.6	23.9
as Shaker packet	2	2.3%	0.0%	2	2.8%	0.0%	-	-
**Non-iodised salt**	**29**	**33.7%**	**4.1%**	**29**	**40.8%**	**4.1%**	**1.3–5.2**	**3.5**
Sea salt	11	12.8%	3.4%	11	15.5%	3.4%	-	-
as Gourmet sea salt	5	5.8%	0.1%	5	7.0%	0.1%	-	-
Rock salt	18	20.9%	0.7%	18	25.4%	0.7%	-	-
as Himalayan salt	16	18.6%	0.7%	16	22.5%	0.7%	-	-
as Shaker packet	6	7.0%	0.0%	6	8.5%	0.0%	-	-
**Salt blends**	**15**	**17.4%**	**0.2%**	**-**	**-**	**-**	**-**	**-**
**Total**	**86**	**-**	**-**	**71**	**-**	**-**	**1.3–45.8**	**23.3**

Notes: ^1^ number of samples (*N*), and their proportion (*N*(%)); ^2^ proportion of total salt sales; ^3^ interval between the lowest and highest determined iodine level, as mg KI/kg of salt; ^4^ sales-weighted iodine levels, calculated as mg KI per kg of salt
